# HDAC3 deteriorates colorectal cancer progression via microRNA-296-3p/TGIF1/TGFβ axis

**DOI:** 10.1186/s13046-020-01720-w

**Published:** 2020-11-17

**Authors:** Jinxiao Li, Man Hu, Na Liu, Huarong Li, Zhaomin Yu, Qian Yan, Minfeng Zhou, Yayuan Wang, Yanjuan Song, Guangtao Pan, Fengxia Liang, Rui Chen

**Affiliations:** 1 Department of Integrated Traditional Chinese and Western Medicine, Union Hospital, Tongji Medical College, Huazhong University of Science and Technology, 1227 Jiefang Avenue, Wuhan City, 430022 Hubei Province China; 2 Rehabilitation Department of traditional Chinese Medicine, Union Red Cross Hospital, Wuhan, 430015 China; 3 Department of oncology, Hubei Provincial Hospital of Integrated Chinese and Western Medicine, Wuhan, 430071 China; 4 First Affiliated Hospital of Guangzhou University of Traditional Chinese Medicine, Guangzhou, 510405 China; 5 College of Acupuncture & Moxibustion and Orthopaedics, Hubei University of Chinese Medicine, Wuhan, 430060 China

**Keywords:** Colorectal cancer, Histone deacetylase 3, MicroRNA-296-3p, Thymine-guanine-interacting factor 1, Transforming growth factor β signaling pathway, Invasion, Migration

## Abstract

**Background:**

The mechanism of histone deacetylase 3 (HDAC3) in colorectal cancer (CRC) has already been discussed. However, the feedback loop of HDAC3/microRNA (miR)-296-3p and transforming growth factor β-induced factor 1 (TGIF1) in CRC has not been explained clearly. Thus, the mainstay of this study is to delve out the mechanism of this axis in CRC.

**Methods:**

To demonstrate that HDAC3 regulates the miR-296-3p/TGIF1/TGFβ axis and is involved in CRC progression, a series of cell biological, molecular and biochemical approaches were conducted from the clinical research level, in vitro experiments and in vivo experiments. These methods included RT-qPCR, Western blot assay, cell transfection, MTT assay, EdU assay, flow cytometry, scratch test, Transwell assay, dual luciferase reporter gene assay, chromatin immunoprecipitation, nude mouse xenograft, H&E staining and TUNEL staining.

**Results:**

Higher HDAC3 and TGIF1 and lower miR-296-3p expression levels were found in CRC tissues. HDAC3 was negatively connected with miR-296-3p while positively correlated with TGIF1, and miR-296-3p was negatively connected with TGIF1. Depleted HDAC3 elevated miR-296-3p expression and reduced TGIF1 expression, decreased TGFβ pathway-related proteins, inhibited CRC proliferation, invasion, and migration in vitro and slowed down tumor growth and induction of apoptosis in vivo, which were reversed by miR-296-3p knockdown. Restored miR-296-3p suppressed TGIF1 and reduced TGFβ pathway-related proteins, inhibited CRC proliferation, invasion, and migration in vitro and slowed down tumor growth and induction of apoptosis in vivo*,* which were reversed by TGIF1 overexpression.

**Conclusion:**

This study illustrates that down-regulation of HDAC3 or TGIF1 or up-regulation of miR-296-3p discourages CRC cell progression and slows down tumor growth, which guides towards a novel direction of CRC treatment.

## Background

Colorectal cancer (CRC) ranks the third most widespread malignancy in the world and the fourth killer for cancer-related mortality [1]. The risk factors mainly consist of inflammatory bowel disease, body mass index, CRC history in first-degree relative and cigarette smoking [2]. At present, bevacizumab and aflibercept are the main anti-angiogenic treatments for metastatic CRC [3]. However, the outcome of pharmacological treatment for CRC currently doesn’t reach the expectancy [4]. Thus, there is an emergency to uncover novel therapies for CRC.

Histone deacetylase (HDAC) enzymes are key regulators of basic cellular events such as cell differentiation, cycle and apoptosis [5]. Of the subfamily, HDAC3 is deregulated in CRC and also serves as a complementary molecular marker for histopathological diagnosis and a prognostic biomarker of CRC [6]. HDAC3 is in part involved in the progression and migration of CRC [7]. Moreover, HDAC3 overexpression is documented to promote CRC proliferation and invasion [8]. MicroRNAs-296-3p (miR-296-3p) serves as a suppressor in malignancies including non-small-cell lung cancer, choroidal melanoma and glioblastoma [9–11]. Widely, it is implied that miR-296 is a suppressor of CRC cell progression and tumor growth [12]. Moreover, miR-296 is also indicative of metastasis and epithelial-mesenchymal transition of CRC [13]. Thymine-guanine-interacting factors (TGIFs) are up-regulated in mutant colon tumors which assist to reprogram the expression of metabolic genes [14]. TGIF1 is a transcriptional co-repressor which exerts as a tumor promoter of CRC exacerbation [15]. Transforming growth factor β (TGFβ) exerts as a pivotal role in the occurrence and metastasis of CRC which functions either as a pro-tumorigenic actor or an anti-tumorigenic actor relying on the stage of the tumor [16]. Inhibition of TGFβ signaling pathway is indicated to inhibit CRC cell progression [17]. There is a study outlining that a HDAC inhibitor in part blocks TGFβ to reduce corneal fibrosis in rabbits [18].

In general, though some studies have illustrated the independent actions of HDAC3, miR-296-3p, TGIF1 and TGFβ pathway in CRC, the interplay among these four factors has been rarely investigated. Based on that, this study is intended to delve out the multilateral interactions among HDAC3, miR-296-3p, TGIF1 and TGFβ signaling pathway in CRC.

## Materials and methods

### Ethics statement

This study was approved and supervised by the ethics committee of Union Hospital, Tongji Medical College Huazhong University of Science and Technology, and the informed consents were obtained from patients.

### Experimental subjects

One hundred twenty-one specimens of CRC tissues and adjacent normal tissues (≥ 5 cm from the tumor margin) were obtained from CRC patients who received treatments in Union Hospital, Tongji Medical College Huazhong University of Science and Technology from January 2012 to November 2018. These patients consisted of 62 males and 59 females, with an average age of 61 years. All specimens had been diagnosed and none of patients had received radiation or chemotherapy before operation. The patients were staged by the tumor node metastasis (TNM) staging standard (the seventh edition) promulgated by Union for International Cancer Control and American Joint Committee on Cancer (AJCC) [19].

### Immunohistochemistry

Sections (3-μm) were dewaxed and hydrated which was followed by antigen retrieval by high-temperature and high-pressure for 3 min. The sections were blocked with 3% peroxidase and probed with the primary antibody HDAC3 (1:2000, Abcam Inc., Cambridge, MA, USA) as well as the secondary antibody. After that, the sections were developed by diaminobenzidine (DAB), which was followed by counterstaining with hematoxylin solution and sealing. A negative control (NC) was set with phosphate buffered saline (PBS) as the primary antibody. Streptavidin-peroxidase staining kit and DAB kit were provided by Beijing Zhongshan Biotechnology Co. Ltd. (Beijing, China). HDAC3 is mainly located in the nucleus [20] and the positive staining shows in brownish yellow. Each sections were evaluated by National Institutes of Health ImageJ software in 5 high-power fields. The ratio of positive cells to total cells in each field was calculated.

### Cell culture

Human normal colonic epithelial cell line FHC and human CRC cell lines SW480, SW620, LOVO and HCT-116 were provided by Shanghai Chinese Academy of Sciences (Shanghai, China). SW480, SW620, LOVO and HCT-116 cells were cultured in dulbecco’s modified eagle medium (DMEM) containing 10% fetal bovine serum (FBS) while FHC cells in DMEM-F12 (Thermo Fisher Scientific, Inc., Waltham, MA, USA) and 10% FBS. All cell lines were incubated (37 °C, 5% CO_2_, saturated humidity) and the medium was renewed every 1–2 d. When the cells reached 80% confluence after 3–5-d incubation, they were passaged at 1:2 or 1:3.

### Cell transfection

SW480 and HCT-116 cells were transfected with their target vectors including HDAC3 siRNA NC, HDAC3 siRNA, HDAC3 siRNA and miR-296-3p inhibitor, miR-296-3p mimic NC, miR-296-3p mimic, TGIF1 siRNA NC, TGIF1 siRNA, and miR-296-3p mimic and pcDNA-TGIF1 (TGIF1 overexpression vector, oe-TGIF1).

Cell transfection: Cells were grew in 6-well plates at 2 × 10^5^ cells/well 36 h earlier before transfection. Upon 60% confluence, the cells were added with the serum-free medium for 1 h. The sequences (100 nmol/L) were transfected into the cells with Lipofectamine 2000 reagent (Invitrogen, Carlsbad, California, USA). All oligonucleotides and plasmids were provided by GenePharma Ltd. Co. (Shanghai, China).

### 3-(4, 5-dimethylthiazol-2-yl)-2, 5-diphenyltetrazolium bromide (MTT) assay

The cells were seeded into 96-well plates at 3 × 10^5^ cells/mL. At 24, 48 and 72 h post cultivation, cells were added with 50 μL MTT solution (5 mg/mL) for 4-h incubation, respectively. An empty group was set without any cells. Subsequently, the culture medium was removed and the cells were reacted with 150 μL dimethyl sulfoxide solution for 10 min. The absorbance (A) value was detected at 490 nm on a microplate reader and 4 duplicates were set for each group. Each reaction was run in triplicate to obtain the average value.

### 5-ethynyl-2-deoxyuridine (EdU) assay

The cells were seeded in 96-well plates at 1 × 10^5^ cells/well for 24 h. Based on the EdU kit (Ribobio, Guangzhou, China), the cells were labeled by EdU and immobilized, which was followed by Apollo and DNA staining. After that, cell proliferation was observed under a fluorescence microscope. The EdU-labeled cells and EdU-unlabeled cells were counted and calculated to obtain the ratio of EdU-labeled cells. Each reaction was run in triplicate to obtain the average value.

### Flow cytometry

Cells were resuspended to the adjusted concentration of 3–6 × 10^6^ cells/mL. The cell suspension (500 μL) was transferred into a 10-mL centrifuge tube by a micropipette and added with 5 mL 70% ice-cold ethanol at 4 °C overnight. Next, the cells were rinsed twice with PBS, centrifuged at 300 g and stained with 500 μL propidium iodide (PI) solution. After that, the cell suspension was filtered through a 400-mesh screen. The cell arrest was detected by flow cytometry. Each reaction was run in triplicate to obtain the average value.

Cells were seeded in 6-well plates at 5.0 × 10^5^ cells/well. The cells were detached with ethylene diamine tetraacetic acid (EDTA)-free 0.25% trypsin, centrifuged, rinsed with pre-cooled PBS and resuspended in 100 μL 1 × Binding Buffer. Cells in the flow tubes in each group were incubated with 5 μL Annexin V-fluorescein isothiocyanate (FITC) and 5 μL PI. Cell cycle and Annexin V-FITC/PI apoptosis detection kits were entrusted to BD Biosciences (Franklin Lakes, NJ, USA). Each reaction was run in triplicate to obtain the average value.

### Scratch test

The cells were seeded in 6-well plates at 4 × 10^5^ cells/well. After transfection, the cells with complete confluence were allowed to draw a straight line along the central axis at an angle perpendicular to the bottom of the 6-well plate by a sterile 10-μL pipette tip. Rinsed with PBS 3 times, the cells were cultured with the serum-free medium for 0.5 h. After that, the cell migration was observed at the 0 h and 48th h under an inverted optical microscope and photographed. Each reaction was run in triplicate to obtain the average value.

### Transwell assay

Trypsinized cells were adjusted to 1 × 10^5^ cells/mL by serum-free culture medium. Three hours before the experiment, a matrigel (BD Bioscience) was incubated in the Transwell chamber (Corning, NY, USA). After that, the solidified matrigel was incubated in the FBS-free culture medium for 40 min. The cell suspension was transferred to the 24-well plate in the Transwell chamber. The outside of the chamber was added with culture medium. After incubation at 37 °C for 48 h, the cells in the Transwell chamber were rinsed with PBS. Cells that did not pass through the membrane were wiped with cotton swabs, while migrated cells were fixed in paraformaldehyde and stained by crystal violet solution. The invasive cells observed under a microscope were counted. Each reaction was run in triplicate to obtain the average value.

### Reverse transcription quantitative polymerase chain reaction (RT-qPCR)

Total RNA extraction from tissues and cells was carried out in conformity with the specifications of Trizol reagent (Tiangen Biotech Co., Ltd., Beijing, China), and RNA concentration and purity were detected by a nucleic acid protein analyzer. RT-qPCR was performed using SYBR premix reagents (Qiagen company, Hilden, Germany). The PCR primers were designed and synthesized by Sangon Biotech Co., Ltd. (Shanghai, China) (Table 1). 2^-△△Ct^ method was adopted to gene expression analysis. Each reaction was run in triplicate to obtain the average value.

**Table 1:** Primer sequences

Genes	Primer sequences
miR-296-3p	F: 5′-GAGGGTTGGGTGGAGGCTCTCC −3′
	The reverse primers of miR-296-3p used universal primers
U6	F: 5′-ATGACACGCAAATTCGTGAAGC-3′
	The reverse primers of U6 used universal primers
HDAC3	F: 5′-GGAGCTGGACACCCTATGAA-3
	R: 5′-TATTGGTGGGGCTGACTCTC-3′
TGIF1	F: 5′-AGATCTGAATTGTGCCAGTGTTTCTCTTTG-3′
	R: 5′-CCATGGCGGCGCTTCAGAGTGAG-3′
TGF-β	F: 5′-GCAAGTGGACATCAACGGGTTC-3′
	R: 5′-CGCACGCAGCAGTTCTTCTC-3′
Smad2	F: 5′-CGTCCATCTTGCCATTCACG-3′
	R: 5′-CTCAAGCTCATCTAATCGTCCTG-3’
Smad3	F: 5′-GGACGCAGGTTCTCCAAAC-3’
	R: 5′-CGGCTCGCAGTAGGTAA-3’
PCNA	F: 5′-CGGATACCTTGGCGCTAGTA-3’
	R: 5′-TCACTCCGTCTTTTGCACAG-3’
Ki-67	F: 5′-GAGAGCTCCCAGCCTAAGGT-3’
	R: 5′-TGCACACCTCTTGACACTCC-3’
MMP2	F: 5′-CCGTCGCCCATCATCAAGTT-3’
	R: 5′-CTGTCTGGGGCAGTCCAAAG-3’
MMP9	F: 5′-CATTTCGACGATGACGAGTTGT-3’
	R: 5′-CGGGTGTAGAGTCTCTCGC-3’
GAPDH	F: 5′-GACAACAGCCTCAAGATCATCAG-3’
	R: 5′-GTGGCAGTGATGGCATGGA-3’

*F* forward, *R* reverse, *miR-296-3p* microRNA-296-3p, *HDAC3* Histone Deacetylase 3, *TGIF1* thymine-guanine-interacting factor1, *TGF-β* transforming growth factor-β, *Smad2* mothers against decapentaplegic homolog 2, *Smad3* mothers against decapentaplegic homolog 3, *PCNA* proliferating cell nuclear antigen, *MMP2* matrix metalloproteinase 2, *MMP9* matrix metalloproteinase 9, *GAPDH* glyceraldehyde-3-phosphate dehydrogenase

### Western blot analysis

The total protein was extracted regarding to the instructions of the protein extraction kit (Thermo Fisher Scientific), and the protein concentration was detected by the bicinchoninic acid protein concentration detection kit (Thermo Fisher Scientific). Electrophoresis was performed on 40 μg protein sample per well. Then, the protein was separated by 10% separation gel and 4% concentrated gel. Next, the protein was transferred to a polyvinylidene fluoride membrane, blocked with 5% skim milk powder, probed with the primary antibodies HDAC3, TGIF1, TGF-β1, phosphorylated (p)-Smad2, p-Smad3 and glyceraldehyde-3-phosphate dehydrogenase (GAPDH) (1:1000, Boster Biological Technology Co. Ltd., Wuhan, Hubei, China) overnight and reprobed with the secondary antibody (1:1500). Finally, the membrane was developed to analyze protein expression with GAPDH as an internal control. Each reaction was run in triplicate to obtain the average value.

### Chromatin immunoprecipitation (ChIP)

The cells were fixed with 1% formaldehyde and quenched with 125 mM glycine. Then, the cells were centrifuged and resuspended in cell lysis buffer (150 mM NaCL, 50 mM Tris pH = 8, 1% Triton X-100, 1% nf-40, 0.01% sodium dodecyl sulfate (SDS), 1.2 mM EDTA pH = 8.0, 1 mM phenylmethylsulfonyl fluoride). The chromatin was broken by ultrasound, and reacted with HDAC3 or immunoglobulin G antibody (Millipore, Massachusetts, USA). Then, the cells were treated with RNase (Qiagen) and proteinase K (Roche, Basel, Switzerland) at 45 °C. DNA was eluted with 100 mM NaHCO_3_ and 1% SDS, and reacted with 300 mM NaCl at 65 °C for 16 h. Qiaquick PCR purification kit (Qiagen) was adopted to purify the immunoprecipitated DNA and the extracted DNA. The purified DNA was amplified by Qiagen QuantiTech SYBR Green PCR master mix in RT-qPCR and subjected to enrichment analysis [21]. Each reaction was run in triplicate to obtain the average value.

### Dual luciferase reporter gene assay

PCR amplification was utilized to generate the 3′-untranslated region (UTR) of TGIF1 containing the binding sites for miR-296-3p. The mutant type (MUT) TGIF1 3′-UTR was constructed by the QuikChange Site-Directed Mutagenesis Kit from Stratagene in the light of manufacturer’s instructions. The obtained TGIF1 3′-UTR sequence and the MUT TGIF1 3′-UTR sequence were inserted into a pmiR-REPORT vector (Promega Corporation, Madison, WI, USA). Then, the WT TGIF1 3′-UTR plasmid and MUT TGIF1 3′-UTR plasmid were transfected with miR-296-3p mimic and its NC into the SW480 cells and HCT-116 cells in compliance with the instructions of Lipofectamine™ 2000 transfection reagent. After 48 h, the samples were collected and the relative luciferase activities of firefly and renilla fluorescence were measured in compliance to the instructions of the dual luciferase detection kit (Promega). Each reaction was run in triplicate to obtain the average value.

### Tumor xenografts in nude mice

Eighty BALB/c nude mice (Wuhan University Laboratory Animal, China), aging 4–5 weeks and weighing 15–19 g, were kept in specific pathogen-free (SPF) animal rooms and randomly divided into groups and injected with the transfected SW480 cells and HCT-116 cells (*n* = 5). The cell suspensions (0.2 mL or 2 × 10^6^ cells) in the logarithmic growth phase were subcutaneously injected into the back of the right forelimb of nude mice under sterile conditions and mice were raised under SPF conditions after injection. The spirit, diet, activity, and defecation of nude mice were observed. The mice were euthanized after 21 d, of which the tumors were dissected to measure the maximum length (a) and width (b) by a vernier caliper. The tumor volume (V) was calculated as (a × b^2^)/2. Part of the tissues were prepared for RT-qPCR and the other part was treated with fixation in formaldehyde, embedment in paraffin and sectioning.

### Hematoxylin-eosin (H&E) staining

Paraffin sections were dewaxed, and the sections were successively placed in absolute ethanol I and II, 95, 80 and 70% ethanol, and double distilled water. Followed by hematoxylin staining and differentiation, the sections were stained with eosin and dehydrated in 70, 80 and 95% ethanol, followed by immersion in absolute ethanol I and II, xylene I and II treatment. The sections were sealed with neutral resin and observed with a microscope.

### Transferase-mediated deoxyuridine triphosphate-biotin nick end labeling (TUNEL) staining

The tissue sections were dewaxed, hydrated and transferred to a dish containing citrate buffer (pH = 6.0). After that, the sections were placed in a microwave oven, irradiated at 350 W for 5 min and rinsed twice in PBS. Next, the sections were immersed in 0.1 mol/L Tris-HCl (pH = 7.5, 3% bovine serum albumin and 30% bovine serum) and reacted with 50 μL TUNEL solution (Roche) in the dark. Also, the sections were reacted with 50 μL converter-peroxidase and then with 50–100 μL DAB for 5–10 min. Finally, the sections were counterstained, dehydrated, sealed and photographed by a microscope.

### Statistical analysis

All data were processed by SPSS 21.0 statistical software (IBM Corp. Armonk, NY, USA). The measurement data were expressed in the form of mean ± standard deviation. Comparisons between two groups were evaluated by t test. Comparisons among multiple groups were evaluated by one-way analysis of variance (ANOVA), followed by Tukey’s multiple comparisons test. The correlations among HDAC3, miR-296-3p and TGIF1 expression in CRC tissues were determined by Pearson correlation analysis. The connection between HDAC3 expression and the clinicopathological characteristics of CRC was identified by Chi-square test. (*) for *P* <  0.05, (**) for *P* <  0.01, and (***) for *P* < 0.001 were indicative of statistical significance.

## Results

### HDAC3 and TGIF1 are highly expressed and miR-296-3p is lowly expressed in CRC tissues and cells

**Fig. 1:**
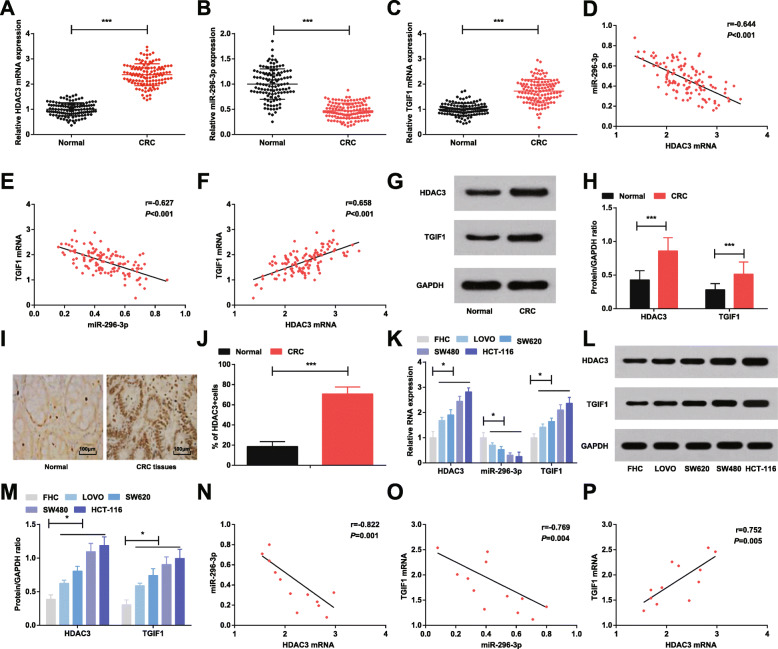
HDAC3 and TGIF1 are highly expressed and miR-296-3p is lowly expressed in CRC tissues and cells. **a**-**c**. RT-qPCR detection of HDAC3, miR-296-3p and TGIF1 expression in CRC tissues and adjacent normal tissues; **d**-**f**. Pearson correlation analysis of the correlations among HDAC3 mRNA, miR-296-3p and TGIF1 mRNA expression in CRC tissues; **g**. Protein bands of HDAC3 and TGIF1 protein in CRC tissues and adjacent normal tissues; **h**. Comparisons of HDAC3 and TGIF1 protein expression in CRC tissues and adjacent normal tissues; **i** & **j**. Representative tissues immunohistochemically stained for HDAC3 in CRC tissues and adjacent normal tissues (× 400) and quantitative analysis of immunohistochemistry of HDAC3; **k**. RT-qPCR detection of HDAC3, miR-296-3p and TGIF1 expression in FHC and CRC cell lines; **l**. Protein bands of HDAC3 and TGIF1 protein in FHC and CRC cell lines; **m**. Comparisons of HDAC3 and TGIF1 protein expression in FHC and CRC cell lines; **n**-**p**. Pearson correlation analysis of the correlation of HDAC3 mRNA, miR-296-3p and TGIF1 mRNA expression in CRC cells. In Fig. **a**-**j**, *n* = 121; In Fig. **k**-**p**, *N* = 3. Comparisons between two groups were evaluated by t test while those among multiple groups by one-way ANOVA, followed by Tukey’s multiple comparisons test. The correlations among HDAC3 mRNA, miR-296-3p and TGIF1 mRNA expression in CRC tissues and cells were analyzed by Pearson correlation analysis. * represented *P* < 0.05, ** represented *P* < 0.01, *** represented *P* < 0.001

The up-regulated HDAC3 has been presented in CRC tissues and silenced HDAC3 disrupts the proliferative, colony-forming and migratory activities, and cell cycle distribution of CRC cells [7]. In order to explore the role of HDAC3 in CRC progression through modulating miR-296-3p/TGIF1 axis, we firstly analyzed the expression of HDAC3, miR-296-3p and TGIF1 in CRC tissues from the level of clinical research. RT-qPCR and western blot analysis were carried out to explain the involved mechanism of HDAC3 in CRC, and findings suggested (Fig. 1a-c, and g, h) that higher expression levels of HDAC3 and TGIF1, and lower expression level of miR-296-3p in CRC tissues. Pearson correlation analysis displayed that HDAC3 mRNA and miR-296-3p expression were negatively connected (*r* = − 0.644, *P* < 0.001), HDAC3 mRNA and TGIF1 mRNA expression were positively connected (*r* = 0.658, *P* < 0.001) and miR-296-3p and TGIF1 mRNA expression were negatively connected (*r* = − 0.627, *P* < 0.001) (Fig. 1d-f).

Immunohistochemistry of HDAC3 expression in CRC tissues and adjacent normal tissues indicated that (Fig. 1i, j) HDAC3 was expressed in the nucleus and more positive cells appeared in CRC tissues.

Based on the mean value of HDAC3 expression, CRC patients were allocated into the high and low expression groups. Further analysis of the connection between HDAC3 expression and clinicopathological characteristics of CRC patients (Table 2) exhibited that higher HDAC3 expression was connected with histological grade, advanced T stage, N stage and AJCC stage (TNM) (all *P* < 0.05).

**Table 2:** Correlation between HDAC3 expression and clinicopathological characteristics of CRC patients

Clinicopathological characteristics	Cases	HDAC3		*P*
	(*n* = 121)	Low expression group (*n* = 52)	High expression group (*n* = 69)	
Age (years)				0.854
≤ 60	54	24	30	
> 60	67	28	39	
Gender				0.463
Male	62	29	33	
Female	59	23	36	
Location				0.530
Left	49	21	28	
Transverse	7	3	4	
Right	37	13	24	
Rectum	28	15	13	
T stage				< 0.001
T1-T2	42	30	12	
T3-T4	79	22	57	
N stage				0.004
N0	58	33	25	
N1-N3	63	19	44	
TNM				0.024
I-II	46	26	20	
III-IV	75	26	49	
Histological grade				0.003
Well	14	12	2	
Moderate	91	34	57	
Poor	16	6	10	

This table uses chi-square test or Fisher’s exact test

In addition, RT-qPCR and western blot analysis (Fig. 1k-m) illustrated that HDAC3 and TGIF1 expression were elevated and miR-296-3p expression was decreased in CRC cell lines by comparison with the FHC cells (all *P* < 0.05).

Also, Pearson correlation analysis mirrored the negative connections between HDAC3 mRNA and miR-296-3p expression (*r* = − 0.822, *P* = 0.001), and miR-296-3p and TGIF1 mRNA expression (*r* = − 0.769, *P* = 0.004), as well as the positive connection between HDAC3 mRNA and TGIF1 mRNA expression (*r* = 0.752, *P* = 0.005) (Fig. 1n-p).

### Knockdown of HDAC3 inhibits CRC cell proliferation and metastasis

**Fig. 2:**
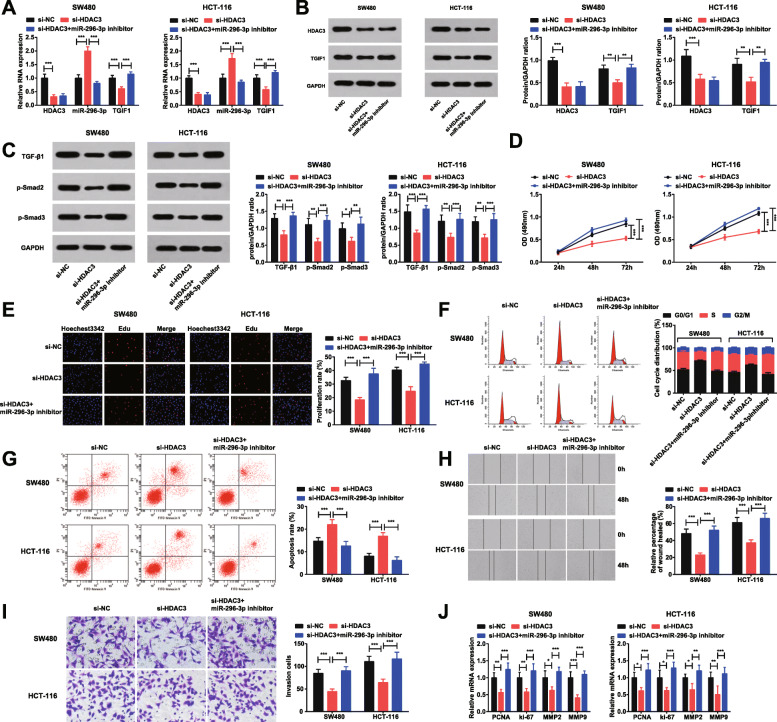
Knockdown of HDAC3 inhibits CRC cell proliferation and metastasis. **a**. RT-qPCR detection of HDAC3 mRNA, miR-296-3p and TGIF1 mRNA expression in cells after knockdown of HDAC3; **b**. Western blot analysis determination of HDAC3 and TGIF1 protein expression in cells after knockdown of HDAC3; **c**. Western blot analysis determination of TGFβ signaling pathway-related proteins in cells after knockdown of HDAC3; **d**. MTT assay of cell viability in cells after knockdown of HDAC3; **e**. EdU assay of cell proliferation after knockdown of HDAC3; **f**. Flow cytometry of cell cycle distribution after knockdown of HDAC3; **g**. Flow cytometry of cell apoptosis after knockdown of HDAC3; **h**. Scratch test of cell migration after knockdown of HDAC3; **i**. Transwell assay of cell invasion after knockdown of HDAC3; **j**. RT-qPCR detection of PCNA, ki-67, MMP2 and MMP9 expression in cells after knockdown of HDAC3. *N* = 3. One-way ANOVA was utilized for comparisons among multiple groups, followed by Tukey’s multiple comparisons test. * represented *P* < 0.05, ** represented *P* < 0.01, *** represented *P* < 0.001. si-NC; HDAC3 siRNA negative control; si-HDAC3; siRNA HDAC3

For the comprehension of the biological effects of HDAC3 in CRC progression, we performed in vitro experiments in CRC cells, and HDAC3 expression was restrained by RNA interference. Moreover, spontaneous knockdown of HDAC3 and transfection of miR-296-3p inhibitor was implemented to explore whether miR-296-3p was involved in HDAC3-mediated functions in CRC. RT-qPCR and western blot analysis (Fig. 2a-c) depicted that si-HDAC3 successfully knocked down HDAC3 expression in cells. After knocking down HDAC3, miR-296-3p was up-regulated while TGIF1 and TGFβ signaling pathway-related proteins were down-regulated. miR-296-3p inhibitor successfully reversed the effects of HDAC3 knockdown on miR-296-3p, TGIF1 and TGFβ signaling pathway-related proteins.

Next, the functions of HDAC3 in CRC cell progression were evaluated by MTT, EdU and Transwell assays, scratch test and flow cytometry (Fig. 2d-i). The results pictured that HDAC3 knockdown decreased OD values, EdU positive cells, invaded cells and wound closure, increased cells arrested in the G0/G1 phase and reduced cells in the S phase, and enhanced apoptosis rate of CRC cells. Moreover, RT-qPCR was adopted to detect the proliferation-related genes PCNA and ki-67, and the metastasis-related genes MMP-2 and MMP-9 in cells after transfection. RT-qPCR revealed that HDAC3 down-regulation decreased PCNA, ki-67, MMP2 and MMP9 expression in CRC cells (Fig. 2j).

Taken together, knockdown of HDAC3 played a suppressive role in CRC cell proliferation, invasion and migration. The underlying mechanism may be related to the regulation of proliferation-related genes PCNA and ki-67 and metastasis-related genes MMP2 and MMP9. Additionally, the functional rescue experiments indicated that down-regulating miR-296-3p reversed the effect of knockdown of HDAC3 on the phenotype of CRC cells, indicating that miR-296-3p is involved in HDAC3-mediated CRC development.

### Restored miR-296-3p or depleted TGIF1 suppresses CRC cell proliferation and metastasis

**Fig. 3:**
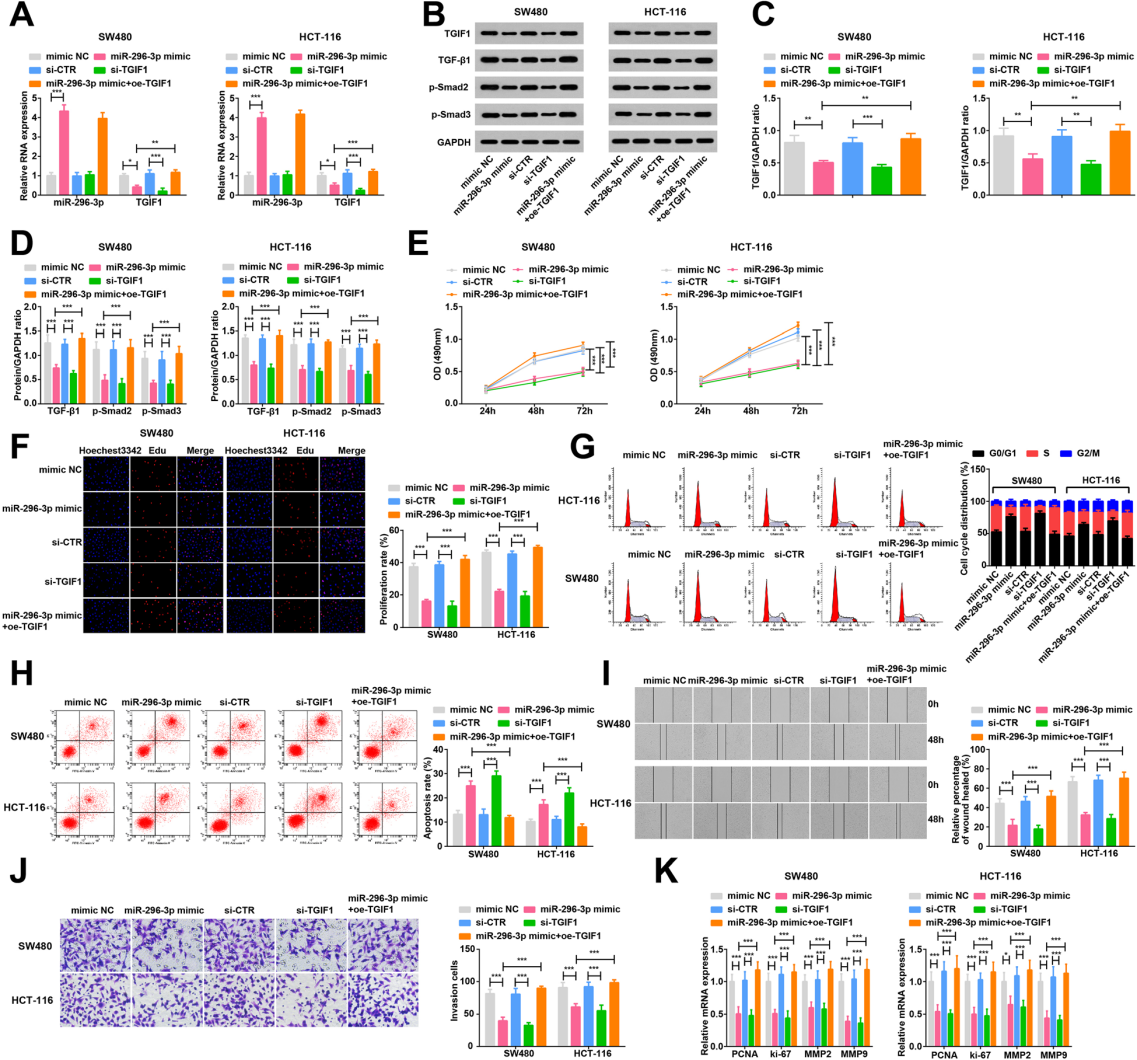
Restored miR-296-3p or depleted TGIF1 suppresses CRC cell proliferation and metastasis. **a**. RT-qPCR detection of miR-296-3p and TGIF1 mRNA expression in cells after up-regulating miR-296-3p or down-regulating TGIF1; **b**. Protein bands of TGIF1 protein in cells after up-regulating miR-296-3p or down-regulating TGIF1; **c**. Comparison of TGIF1 protein expression in cells after up-regulating miR-296-3p or down-regulating TGIF1; **d**. Analysis of TGFβ signaling pathway-related proteins in cells after up-regulating miR-296-3p or down-regulating TGIF1; **e**. MTT assay of cell viability after up-regulating miR-296-3p or down-regulating TGIF1; **f**. EdU assay of cell proliferation after up-regulating miR-296-3p or down-regulating TGIF1; **g**. Flow cytometry of cell cycle distribution after up-regulating miR-296-3p or down-regulating TGIF1; **h**. Flow cytometry of cell apoptosis after up-regulating miR-296-3p or down-regulating TGIF1; **i**. Scratch test of cell migration after up-regulating miR-296-3p or down-regulating TGIF1; **j**. Transwell assay of cell invasion after up-regulating miR-296-3p or down-regulating TGIF1; **k**. RT-qPCR of PCNA, ki-67, MMP2 and MMP9 expression after up-regulating miR-296-3p or down-regulating TGIF1. N = 3. One-way ANOVA was utilized for comparisons among multiple groups, followed by Tukey’s multiple comparisons test. * represented *P* < 0.05, ** represented *P* < 0.01, *** represented *P* < 0.001. si-CTR; TGIF1 siRNA negative contro; si-TGIF1: siRNA TGIF1; oe-TGIF1: TGIF1 overexpression vector

Then, for exploration of the impacts of miR-296-3p and TGIF1 on CRC proliferation and metastasis, CRC cells were transfected with miR-296-3p mimic or si-TGIF1. Western blot analysis and RT-qPCR verified the successful regulation of miR-296-3p and TGIF1 expression in cells. miR-296-3p restoration or TGIF1 depletion decreased TGFβ signaling pathway-related protein expression. Moreover, Overexpressing TGIF1 reversed the effect of miR-296-3p up-regulation on TGFβ signaling pathway-related protein (Fig. 3a-d).

Subsequently, the roles of miR-296-3p and TGIF1 in CRC proliferation and metastasis were disclosed. Various assays manifested that restoration of miR-296-3p or depletion of TGIF1 reduced OD values, EdU positive cells, invasive cells and wound closure, increased cells in the G0/G1 phase, decreased cells in the S phase and elevated apoptosis rate (Fig. 3e-j), and reduced PCNA, ki-67, MMP2 and MMP9 expression (Fig. 3k). The functional rescue experiments revealed that TGIF1 elevation offset the cell biological changes caused by miR-296-3p restoration, hinting that miR-296-3p disturbed CRC proliferation and metastasis via suppressing TGIF1.

### HDAC3 up-regulates TGIF1 via miR-296-3p inhibition

**Fig. 4:**
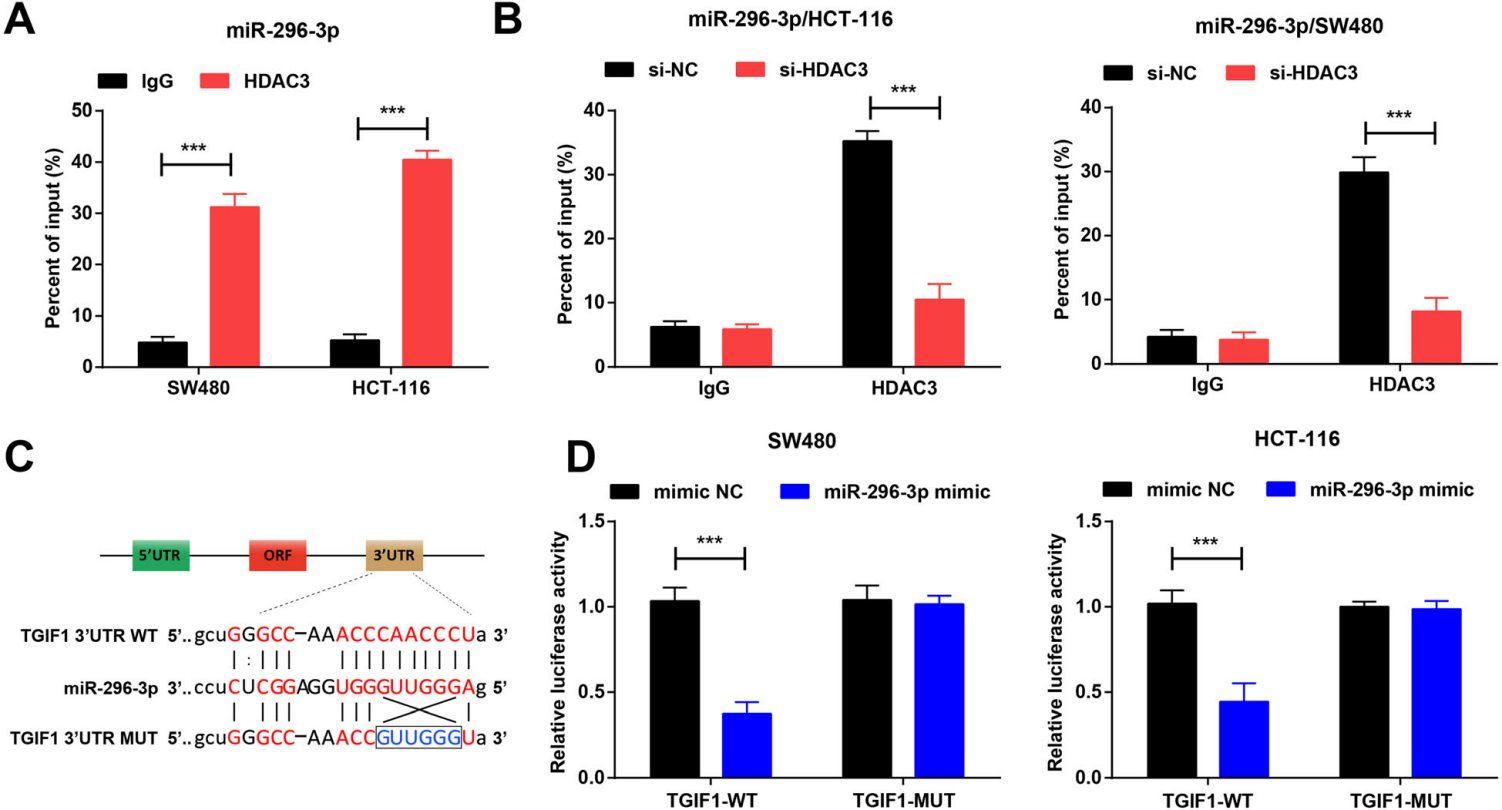
HDAC3 up-regulates TGIF1 via miR-296-3p inhibition. **a**. ChIP-qPCR detection of the recruitment level of HDAC3 in the miR-296-3p promoter; **b**. ChIP-qPCR detection of the recruitment level of HDAC3 in the miR-296-3p promoter after HDAC3 knockdown; **c**. The putative binding sites of miR-296-3p and TGIF1 were predicted and mutated for the following assays; **d**. Dual luciferase reporter gene assay of the targeting relationship between miR-296-3p and TGIF1. *N* = 3. The comparisons between two groups were analyzed by t test. * represented *P* < 0.05, ** represented *P* < 0.01, *** represented *P* < 0.001. WT: wild-type; MUT: mutant

The above assays illustrated that miR-296-3p participated in the HDAC3-mediated functions in CRC cell proliferation and metastasis and knocking down HDAC3 raised miR-296-3p expression. Therefore, it was speculated that a binding relationship existed between HDAC3 and miR-296-3p. ChIP assay confirmed that HDAC3 bound to miR-296-3p promoter (Fig. 4a). HDAC3 low expression vector reduced the recruitment level of HDAC3 on the miR-296-3p promoter (Fig. 4b).

The potential target genes of miR-296-3p were predicted by prediction tools (starBase and RNA22). As a candidate gene of miR-296-3p, TGIF1 (Fig. 4c) was opted because of its influences on cancers [22–24]. To verify the targeting relation between those two, the reporters of TGIF1-WT and TGIF1-MUT containing miR-296-3p binding site were produced. These two types of reporters were severally co-transfected with miR-296-3p mimic or its NC into SW480 or HCT-116 cells. Luciferase activity detection presented that miR-296-3p mimic had no effect on the luciferase activity in the TGIF1-MUT reporter but impaired that in the TGIF1-WT (Fig. 4d). It was implied that HDAC3 up-regulated TGIF1 by inhibiting miR-296-3p.

### Restored miR-296-3p or depleted TGIF1/HDAC3 slows down tumor growth in nude mice with CRC

For further confirmation of the attended role of miR-296-3p/TGIF1/HDAC3 axis in CRC, in vivo experiments were performed and the transfected SW480 and HCT-116 cells were injected into the nude mice. On the 21st d of injection, the tumors were weighed and measured. Then, the tumors were utilized for detecting TGF-β1, Smad2 and Smad3 expression and H&E staining and TUNEL staining.

**Fig. 5:**
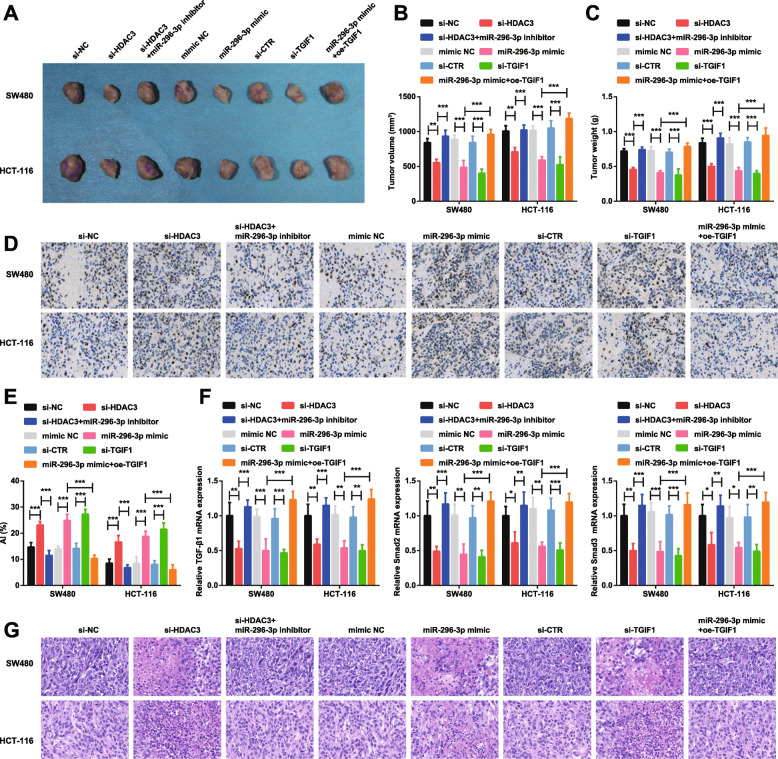
Restored miR-296-3p or depleted TGIF1/HDAC3 slows down tumor growth in nude mice with CRC. **a**. Representative images of xenografted tumors in nude mice in each group; **b**. Volumes of xenografted tumors in nude mice in each group; **c**. Weights of xenografted tumors in nude mice in each group; **d**. TUNEL staining (× 200) of xenografted tumor tissues; **e**. Apoptotic index of xenografted tumor tissues in nude mice in each group; **f**. RT-qPCR detection of TGF-β1, Smad2 and Smad3 mRNA expression in xenografted tumor tissues in nude mice in each group; **g**. HE staining of xenograft tumor tissues in each group (× 200). *n* = 5. One-way ANOVA was utilized for comparisons among multiple groups, followed by Tukey’s multiple comparisons test. * represented *P* < 0.05, ** represented *P* < 0.01, *** represented *P* < 0.001

The results validated that down-regulation of HDAC3 or TGIF1, or up-regulation of miR-296-3p resulted in suppressed tumor growth (Fig. 5a-c), elevated apoptosis rate (Fig. 5d, e) and inhibited TGF-β1, Smad2 and Smad3 expression (Fig. 5f). In addition to that, the CRC tumor cells were abundant and arranged disorderly, showing different sizes and shapes, nuclear atypia, and pathological mitosis. After depletion of HDAC3 or TGIF1 or augment of miR-296-3p, the tumor cells were characterized by cellular shrinkage, dense chromatin in the nucleus, markedly reduced mitotic division, with scattered foci and necrotic lesions fused into sheets (Fig. 5g). The rescue experiments suggested that down-regulated miR-296-3p mitigated depleted HDAC3-induced effects, and overexpressed TGIF1 abrogated up-regulated miR-296-3p-induced effects.

## Discussion

Primary CRC is a malignant tumor that originates in the colon or rectum [25]. HDAC3 is indicated in the pathogenesis and deterioration of CRC [6]. However, the interplay among HDAC3, miR-296-3p, TGFβ signaling pathway and TGIF1 needs sufficient expedition. Hence, the study is intended to probe into the mechanism translation of HDAC3, miR-296-3p, TGFβ signaling pathway and TGIF1 in CRC and it is elucidated that HDAC3 deteriorates CRC progression and promotes tumor growth via miR-296-3p/TGIF1/TGFβ axis.

To begin with, the expression of HDAC3, miR-296-3p and TGIF1 in CRC tissues and cells is determined with the finding demonstrating higher HDAC3 and TGIF1 and lower miR-296-3p expression levels in CRC tissues and cells. Lately, HDAC3 is manifested to be up-regulated in tissues of CRC [7]. Actually, it is previously described that HDAC3 is highly expressed in CRC [6]. Supported by a former study, the expression of TGIF1 is lower than the basic level in CRC [26]. Additionally, another study has also been conducted with the result illustrating an increment in the expression of TGIF1 in CRC [15]. In the light of miR-296-3p expression, there is a study revealing the lower miR-296-3p expression in patient with CRC [12]. Mechanistically, miR-296 expression trends towards a reduction in CRC tissues and cells [13]. Collectively, the expression of HDAC3, miR-296-3p and TGIF1 is in compliance with previous studies.

Subsequently, experiments of HDAC3 and TGIF1 knockdown and miR-296-3p up-regulation are performed to identify their roles in CRC cell progression and it is depicted that either reduction of HDAC3 or TGIF1 or up-regulation of miR-296-3p is suppressive in CRC cell proliferation and metastasis. There is an advanced study depicting that down-regulation of HDAC3 by si-HDAC3 serves as an inhibitor in the CRC cell proliferation, colony-forming ability and migration [7]. Similarly, depleted HDAC3 is believed to inhibit hepatocyte proliferation in hepatocellular carcinoma [27]. In addition, a former study has highlighted that the suppression of metastasis of CRC cells may be ascribed to silencing of HDAC3 [28]. A reduction in the TGIF expression is commonly suggested a decrease in the cell proliferation and metastasis in some types of cancer such as breast cancer and lung adenocarcinoma [29, 30]. Notably, the depletion of TGIF works as the disturbance of proliferation and tumorigenesis of esophageal cancer EC109 cells and A549 cells [24, 31]. Currently, it is manifested that incremental miR-296 restricts cell growth and promotes cell apoptosis in CRC [12]. Besides that, restoration of miR-296 results in suppressed proliferation and invasion in cervical cancer [32].

Except the in vitro experiments in CRC cells, the functions of HDAC3, TGIF1 and miR-296-3p in CRC are further verified by in vivo experiments in nude mice. It is demonstrated that down-regulated HDAC3 or TGIF1 or up-regulated miR-296-3p is the restriction for tumor growth in nude mice. As described in advance, the HDAC3 inhibitor, RGFP966 plays an inhibitory actor for tumor growth in hepatocellular carcinoma [33]. Moreover, inhibited xenografted tumor growth in liver cancer is partially ascribed to HDAC3 inhibition [27]. Additionally, the suppression of HDAC3 is proved to discourage xenografted tumor growth in prostate cancer [34]. Interestingly, restoring miR-296 is surveyed to take part in the tumor growth restriction in breast cancer [35]. Lastly, this study has stated that HDAC3 upregulates TGIF1 via miR-296-3p inhibition. There is a study manifesting that TGIF recruits HDACs and plays a role in determining the effect of TGFβ1 on microglia [36]. Moreover, down-regulation of HDAC4 stimulates the expression of TGIF and TGIF2 homeoproteins, which are endogenous repressors of the TGFβ signaling pathway [37]. However, the more concrete interplay among HDAC3, TGIF1 and miR-296-3p should be comprehensively explored.

## Conclusion

On the whole, the present study has pictured the mechanisms of HDAC3, TGIF1, TGFβ signaling pathway and miR-296-3p in CRC that knockdown of HDAC3 or TGIF1 or up-regulation of miR-296-3p blocks CRC development and tumor growth via inhibiting TGFβ signaling pathway. However, in-depth studies are still in requirement in a larger cohort.

## Data Availability

Not applicable.

## Abbreviations

HDAC3: Histone deacetylase 3
CRC: Colorectal cancer
TGIF1: Transforming growth factor β-induced factor 1
HDAC: Histone deacetylase
TGIFs: Thymine-guanine-interacting factors
TGFβ: Transforming growth factor β
TNM: Tumor node metastasis
AJCC: American Joint Committee on Cancer
DAB: Diaminobenzidine
NC: Negative control
PBS: Phosphate buffered saline
DMEM: Dulbecco’s modified eagle medium
FBS: Fetal bovine serum
PI: Propidium iodide
EDTA: Ethylene diamine tetraacetic acid
FITC: Fluorescein isothiocyanate
RT-qPCR: Reverse transcription quantitative polymerase chain reaction
GAPDH: Glyceraldehyde-3-phosphate dehydrogenase
ChIP: Chromatin immunoprecipitation
SDS: Sodium dodecyl sulfate
UTR: Untranslated region
MUT: Mutant type
SPF: Specific pathogen-free
H&E: Hematoxylin-eosin
ANOVA: One-way analysis of variance
