# Correction to: HDAC3 deteriorates colorectal cancer progression via microRNA-296-3p/TGIF1/TGFβ axis

**DOI:** 10.1186/s13046-021-02232-x

**Published:** 2021-12-31

**Authors:** Jinxiao Li, Man Hu, Na Liu, Huarong Li, Zhaomin Yu, Qian Yan, Minfeng Zhou, Yayuan Wang, Yanjuan Song, Guangtao Pan, Fengxia Liang, Rui Chen

**Affiliations:** 1 Department of Integrated Traditional Chinese and Western Medicine, Union Hospital, Tongji Medical College, Huazhong University of Science and Technology, 1227 Jiefang Avenue, Wuhan City, 430022 Hubei Province China; 2 Rehabilitation Department of traditional Chinese Medicine, Union Red Cross Hospital, Wuhan, 430015 China; 3 Department of Oncology, Hubei Provincial Hospital of Integrated Chinese and Western Medicine, Wuhan, 430071 China; 4 First Affiliated Hospital of Guangzhou University of Traditional Chinese Medicine, Guangzhou, 510405 China; 5 College of Acupuncture & Moxibustion and Orthopaedics, Hubei University of Chinese Medicine, Wuhan, 430060 China

**Correction to: J Exp Clin Cancer Res 39, 248 (2020)**

**https://doi.org/10.1186/s13046-020-01720-w**

Following publication of the original article [1], the authors identified some minor errors in Figure 1, specifically:

- Fig. 1i: incorrect images used for immunohistochemical tests (Normal and CRC tissues); the images have been replaced

**Fig. 1:**
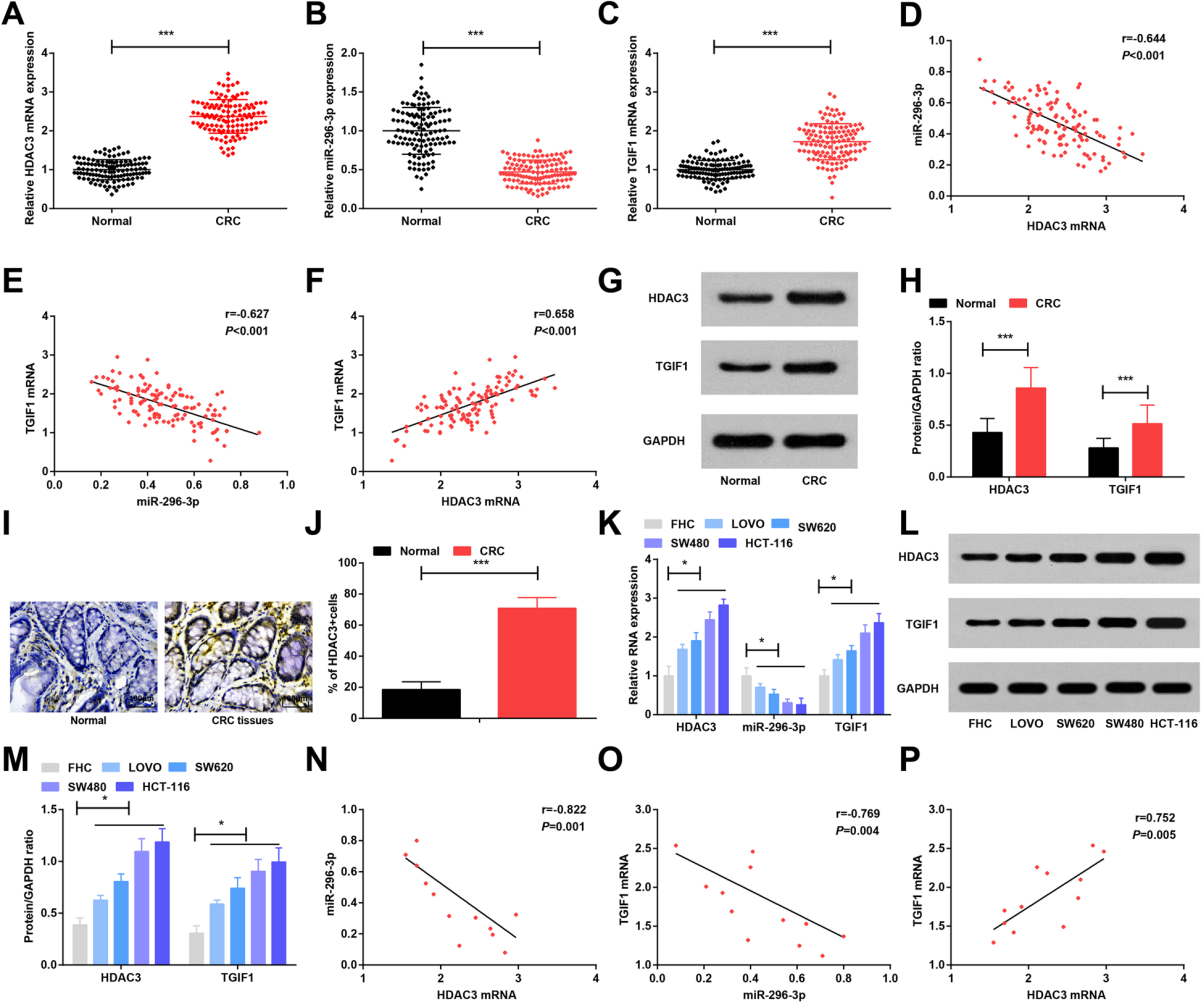
HDAC3 and TGIF1 are highly expressed and miR-296-3p is lowly expressed in CRC tissues and cells. **a-c**. RT-qPCR detection of HDAC3, miR-296-3p and TGIF1 expression in CRC tissues and adjacent normal tissues; **d-f**. Pearson correlation analysis of the correlations among HDAC3 mRNA, miR-296-3p and TGIF1 mRNA expression in CRC tissues; **g**. Protein bands of HDAC3 and TGIF1 protein in CRC tissues and adjacent normal tissues; **h**. Comparisons of HDAC3 and TGIF1 protein expression in CRC tissues and adjacent normal tissues; **i** & **j**. Representative tissues immunohistochemically stained for HDAC3 in CRC tissues and adjacent normal tissues (× 400) and quantitative analysis of immunohistochemistry of HDAC3; **k**. RT-qPCR detection of HDAC3, miR-296-3p and TGIF1 expression in FHC and CRC cell lines; **l**. Protein bands of HDAC3 and TGIF1 protein in FHC and CRC cell lines; **m**. Comparisons of HDAC3 and TGIF1 protein expression in FHC and CRC cell lines; **n-p**. Pearson correlation analysis of the correlation of HDAC3 mRNA, miR-296-3p and TGIF1 mRNA expression in CRC cells. In Fig. **a-j**, *n* = 121; In Fig. **k-p**, *N* = 3. Comparisons between two groups were evaluated by t test while those among multiple groups by one-way ANOVA, followed by Tukey’s multiple comparisons test. The correlations among HDAC3 mRNA, miR-296-3p and TGIF1 mRNA expression in CRC tissues and cells were analyzed by Pearson correlation analysis. * represented *P* < 0.05, ** represented *P* < 0.01, *** represented *P* < 0.001

The corrected figure is given here. The correction does not have any effect on the final conclusions of the paper. The original article has been corrected.
